# Supplementary material for: HNRNPL Restrains miR-155 Targeting of BUB1 to Stabilize Aberrant Karyotypes of Transformed Cells in Chronic Lymphocytic Leukemia
Source: Cancers (Basel). 2019 Apr 23;11(4):575. doi: 10.3390/cancers11040575 (PMC6520824; doi:10.3390/cancers11040575)
Supplement: Supplementary file 1 [file cancers-11-00575-s001.pdf]

Supplementary Material: HNRNPL Restrains *miR-155* Targeting of BUB1 to Stabilize Aberrant Karyotypes of Transformed Cells in Chronic Lymphocytic Leukemia

Sara Pagotto, Angelo Veronese, Alessandra Soranno, Veronica Balatti, Alice Ramassone, Paolo E. Guanciali-Franchi, Giandomenico Palka, Idanna Innocenti, Francesco Autore, Laura Z. Rassenti, Thomas J. Kipps, Renato Mariani-Costantini, Luca Laurenti, Carlo M. Croce and Rosa Visone

Table S1. Karyotype of $HDF_{LT/hTERT}$ cells at passages 16, 20 and 24.

	$HDF_{LT/hTERT}$		
**Metaphases**	**P16**	**P20**	**P24**
1	46, XX, del (4q), del (Xq)	45, X, −X	45, XX, del (Xq), −2, der (17)
2	46, XX, del (2q), add (X)	47, XX, +7	47, XX, +7, del (5p)
3	46, XX, del (7q), t (7;9)	45, XX, −2	47, XX, +mar
4	46, XX, add (14)	46, XX	47, XX, +7
5	46, XX,	46, XX	47, XX, +7
6	46, XX, del (14q)	46, XX	46, XX
7	46, XX,	46, XX	45, XX, −13
8	46, XX, del (2p)	46, XX	44, XX, −13, −14
9	43, XX, −7, −8, −18, −19, +mar	46, XX	46, XX, +7, iso (5q), −10
10	46, XX,	47, XX, +7	46, XX, del (7q)
11	46, XX, +7, −6	45, X, −X, −11, −22, +15, +16	46, XX, −14, der (5), +mar
12	45, X, −X	47, XX, +7	46, XX
13	45, XX, −13	46, XX	45, XX, −15
14	45, XX, −8, add (19)	91	46, XX
15	45, X, −X, del (7p)	47, XX, +7	46, XX, del (5q)
16	44, XX, −2, −12, del (1q), del (Xq)	46, XX	44, XX, del (Xq), del (9q), del (2p), −13, −14
17	46, XX	45, XX, −2	47, XX, +7, del (4p), del (5p)
18	47, XX, +7, del (5p)	46, XX	47, XX, +7, del (20)
19	46, XX	92	44, XX, −13, −14
20	47, XX, +7, del (5p)	45, XX, −15, del (2q)	45, XX, −8
21	46, XX	44, X, −X, −22	44, X, −X, −4
22	46, XX	46, XX	90
23	45, XX, add (11p), add (18), −8	92	46, XX, add (5)
24	46, XX	47, XX, +7	45, XX, −12, der (17), t (17;12)
25	47, XX, +7, del (5p)	47, XX, +7	46, XX
26	46, XX	47, XX, +7, del (5p)	47, XX, +7
27	47, XX, +7	46, XX	94
28	48, XX, +7, +mar	45, XX, −2	92
29	47, XX, +7	45, X, −X	46, X, −X, +mar, del (6q)
30	46, XX	46, XX, −2, +7	46, XX
31	46, XX, del (4p), der (20)	46, XX	45, XX, −13, del (6q)
32	46, XX	45, XX, −2	45, XX, −4, add (22), t (4;22)
33	45, XX, −4	45, X, −X, der (3)	45, XX, −21
34	46, XX	45, XX, −15, del (5p)	45, XX, −4, der (5)
35	46, XX	45, XX, −2	45, XX, −22
36	45, XX, −2	45, XX, −13	45, XX, −14
37	47, XX, +7	44, X, −X, −10, t (15;10)	45, XX, +mar, −8

38	45, XX, −13	46, XX	46, XX, del (7q)
39	43, XX, −8, −11, −18, add (18q)	46, XX, del (17q)	46, XX
40	46, XX, del (5q)	47, XX, +7, del (5p), der (13)	47, XX, +7
41	45, XX, −2	46, XX	45, XX, −8
42	44, XX, −13, −14	46, XX	44, XX, −15, −21, add (7)
43	46, XX	46, XX	46, XX, −2, +mar, t (14;22)
44	46, XX	45, XX, −13	46, XX, +7, −14, add (5), t (5;14)
45	46, XX, +7, −6, t (5;6)	47, XX, +7	46, XX, +7, −14
46	45, XX, −17	90	45, X, −X
47	46, XX, −5, −12, del (6q)	46, XX	46, XX, del (15q)
48	46, XX, −2, −19, +2mar	46, XX	46, XX
49	45, X, −X	46, XX del (4p), der (22)	46, XX, −13, +7
50	46, XX, −2, +7	44, XX, −13, −16	46, XX
51	46, XX	45, XX, −2	46, XX
52	45, XX, −4	46, XX	46, XX, del (Xq), add (4), der (16)
53	46, XX, del (Xq)	46, XX	45, XX, −7, add (5), der (6)
54	47, XX, +7, del (5p), del (11q)	47, XX, +7	46, XX, del (17p)
55	45, XX, −4, t (2;14)	46, XX	44, X, −X, −14, der (19)
56	47, XX, +7, t (2;7)	47, XX, +7	47, XX, +7
57	46, XX, del (4q)	46, XX, del (11p)	45, XX, −15
58	46, XX	92	46, XX
59	45, X, −4, −X, +mar	46, XX	45, XX, −13
60	45, X, −X	46, XX	46, XX, +7, del (8p), add (5), t (5;15), −15
61	47, XX, +7, del (5p)	44, XX, −10, −13, −15, +mar	46, XX, −2, +mar
62	47, XX, +2mar	46, X, −X, −8, +7, +mar, del (5p)	91
63	46, XX	45, XX, −13	45, XX, −4
64	46, XX	46, XX, −13, del (7p), del (9q), +mar	47, XX, +7
65	46, XX	90	47, XX, +7, +9, del (1q)
66	45, XX, −4	47, XX, +7	45, XX, −21
67	46, XX, t (5;22)	47, XX, +7	46, XX
68	47, XX, del (5p), +7	46, XX	46, XX
69	47, XX, +7, del (5p)	46, XX	45, XX, −19
70	46, XX	47, XX, +7	45, XX, −13
71	47, XX, +7, del (5p)	47, XX, +7, del (5p)	45, XX, −4
72	46, XX, add (10q)	44, XX, −13, −18, del (17q)	45, XX, −22
73	46, XX	46, XX	46, XX
74	46, XX, del (5q)	46, XX	48, XX, −13, + 3mar, del (Xq)
75	46, XX	47, XX, +mar	46, XX
76	45, X, −X	45, XX, −8, del (1q), der (15)	46, XX
77	46, XX	46, XX	46, XX, +4, −8, add (22)
78	46, XX, iso (21q)	46, XX	44, XX, −9, −15
79	46, XX	47, XX, +2	46, XX, +7, −14, t (4;14)
80	46, XX, del (5q)	46, XX, del (3p)	47, XX, +7
81	47, XX, +7, del 5p	46, XX	45, XX, −13
82	46, XX	46, XX	46, XX, add (4), add (17), del (Xq)
83	46, XX, del (7q)	46, XX, der (4), del (17p)	44, XX, −4, −14
84	46, XX	46, XX, del (2q)	46, XX
85	46, XX	46, XX, del (1q)	46, XX, del(4p), add (21)
86	45, XX, −14, −19, +7	45, X, −X, der (13)	46, XX
87	43, XX, −11, −16, −22, t (14;22), del(17p)	46, XX	46, X, −11, +14, −X, +21
88	44, XX, −8, −11, add (19)	46, X, −X, + 2mar	45, XX, −1
89	44, XX, −8, −14	45, XX, −19, add (5)	47, XX, +7, t (5;6), −5, +8

90	46, XX	46, XX	46, XX
91	46, XX	47, XX, +7	46, XX
92	45, X, −X	46, XX, del (2p)	45, X, −X, −4, +15, add (19)
93	46, XX	45, XX, del (6q), +13, −15, −19	44, X, −X, −20, der (13)
94	46, XX	46, XX, del (2q)	
95		47, XX, +7	

Table S2. Genetic, molecular and clinical features of the B-CLL patients.

ID	Sex	Sampling Data (Day/Month/Year)	MBL/CLL	Stage	IGHV (Mutated/ Unmutated)	WBCs (Cells/mL)	CD5/CD19 (% in PBMC)	Therapy Need
**LLC05**	Male	18/05/2011	CLL	BII	mut	50000	89	no
		22/05/2013	CLL	BII		88000	85.4	no
**LLC21**	Male	11/10/2011	MBL	A0	mut	15800	40.7	no
		20/01/2016	CLL	A0		25400	80.9	no
**LLC31**	Male	29/11/2011	CLL	A0	mut	18690	71.3	no
		20/05/2015	CLL	A0		21400	74.5	no
**LLC34**	Male	06/12/2011	MBL	A0	mut	8500	20.6	no
		30/10/2012	CLL	BII		24170	89.1	no
		28/05/2013	CLL	BII		37710	90.6	no
		09/04/2014	CLL	CIV		56340	83.2	si
**LLC47**	Female	14/02/2012	MBL	A0	mut	15290	35.6	no
		20/01/2016	CLL	BII		24500	64.6	no
**LLC52**	Male	06/03/2012	CLL	AII	mut	29100	92.4	no
		24/07/2012	CLL	AII		22600	89.7	no
		19/03/2013	CLL	BII		33000	91.9	no
**LLC62**	Male	31/07/2012	CLL	A0	unmut	23950	76.2	no
		11/09/2012	CLL	BI		29710	80.5	no
		04/06/2013	CLL	BI		49000	87.7	no
**LLC70**	Female	09/10/2012	CLL	BII	unmut	72700	91.5	no
		06/02/2013	CLL	BII		134000	91.1	si
**LLC76**	Male	18/12/2012	CLL	BII	mut	48360	69.3	no
		19/03/2014	CLL	BII		48400	82.2	si
**LLC81**	Female	29/01/2013	CLL	BII	mut	72000	86.2	no
		12/02/2014	CLL	BII		113600	93.5	si
**LLC87**	Female	28/05/2013	CLL	A0	mut	43400	91.2	no
		12/11/2014	CLL	A0		37200	85.3	no
**LLC98**	Male	27/02/2014	CLL	BII	unmut	38660	84.15	no
		18/06/2014	CLL	BII		85990	89.53	si
**LLC108**	Female	15/10/2014	CLL	BII	mut	52300	73.6	no
		26/03/2015	CLL	BII		109000	89.5	si

MBL, monoclonal B-cell lymphocytosis; CLL, chronic lymphocytic leukemia; IGHV, immunoglobulin heavy chain variable-region; WBCs, white blood cells; PBMC, peripheral blood mononuclear cells; CD5, CD5 molecule; CD19 molecule.

Table S3. Oligonucleotide primers used in the study for microRNA expression (miRE), mutational analysis (MA), reverse transcription (RT) and gel electrophoresis (Gel). The sequences of the UPLs-Locked Nucleic Acids (Universal Probe library, Roche) used for qPCR are indicated. FAM is the 6-carboxyfluorescein dye and Q is the fluorescein quencher.

Name	Forward Primer	Reverse Primer	Probe/ Assay	Method
BUB1-ex-2-3	GACTATGTCTGAGCGAATGC	AGTGGCACAGAAGTTCGTTG		MA
BUB1-ex-4	CAGACATAGACTAGCAGTTCC	TACCCACACAGGTACAGGAA		MA
BUB1-ex-24	CTAAACTGTCCAAGCCTGTG	AGGGACCTTATGGGGTTGTA		MA
BUB1-ex-7-8	CTACTTGGGATTAAGGTCTC	TCTGGGCTTCTGATAGGATG		MA
BUB1-ex-12	CTGAGTGTGTAGCACCTGAT	CATTCCCTGCCTTTATGTGAC		MA
BUB1-ex-14-15	CATGTAGTAGCCAAGCTGTG	CACAATATCCTCTGACTGGCA		MA
BUB1-ex-20-21	CGTGCAAGGTACAAGCCAAA	CAACTGCAGACAGTGGATGC		MA
BUB1-ex-22-23	TCTCCTTCAACTGCTCCTTA	TTTGGCTTGTACCTTGCACG		MA
BUB1-ex-24	CAGATGGAACCCAACATTCTAC	CTTGCTGCCTAAGTTAGACAG		MA
BUB1-ex-16	TTGTGGGCTGTATTAGCCAG	TCCCATGTGGAATTTCCATG		MA
BUB1-ex-17	ACGGAGAGTACCTTCTGACT	ATGGTGTGTGTTGTGCACAC		MA
BUB1-ex-6	TCTGAGACAGTGTGAATGAG	ATGGAGCTCAGAGCCATGAA		MA
BUB1-ex-10	AGACAGATGCCATGCTTTGA	CTGTTCCTGATAATGCAGGTC		MA

BUB1-ex-5	ACTTGACACGTGGAGAAGAG	AAGTGCTGGGATTACAGGTG		MA
BUB1-ex-9	CATGTAGTAGCCAAGCTGTG	CAAACTGCCATTCCTGCTGC		MA
BUB1_3569-4090	CTGCTCTTAGAATGTAAGCG	GAACTGTGCATAACCTGGGA		Gel elect.
U58_F	ACTTTTTAAACACTGACCTTCC			Gel
U64_ACTB (97 bp)	CCAACCGCGAGAAGATGA	CCAGAGGCGTACAGGGATAG		Gel
ACTB (593 bp)	AGAAAATCTGGCACCACACC	AGGAAGGAAGGCTGGAAGAG		Gel
RT_155	GTTGGCTCTGGTGCAGGGTCCGAGGTATTCGCACCAGAGCCAACAC CCCT			RT
miR-155	GCGGCGGTTAATGCTAATCGTG	GTGCAGGGTCCGAGGT	UPL21/ Fam-Q	miRE
RT_U44	GTTGGCTCTGGTGCAGGGTCCGAGGTATTCGCACCAGAGCCAACA GTCAGTT			RT
U44	GCGGCGGCCTGGATGATGATAG	GTGCAGGGTCCGAGGT	UPL21/ Fam-Q	miRE
D2S1888 (6FAM)	[6FAM] TTTGAAGTTTGGTGTCTGTGTAA	GTCCCTTGGAAATGTTAGGG	FAM	FLA

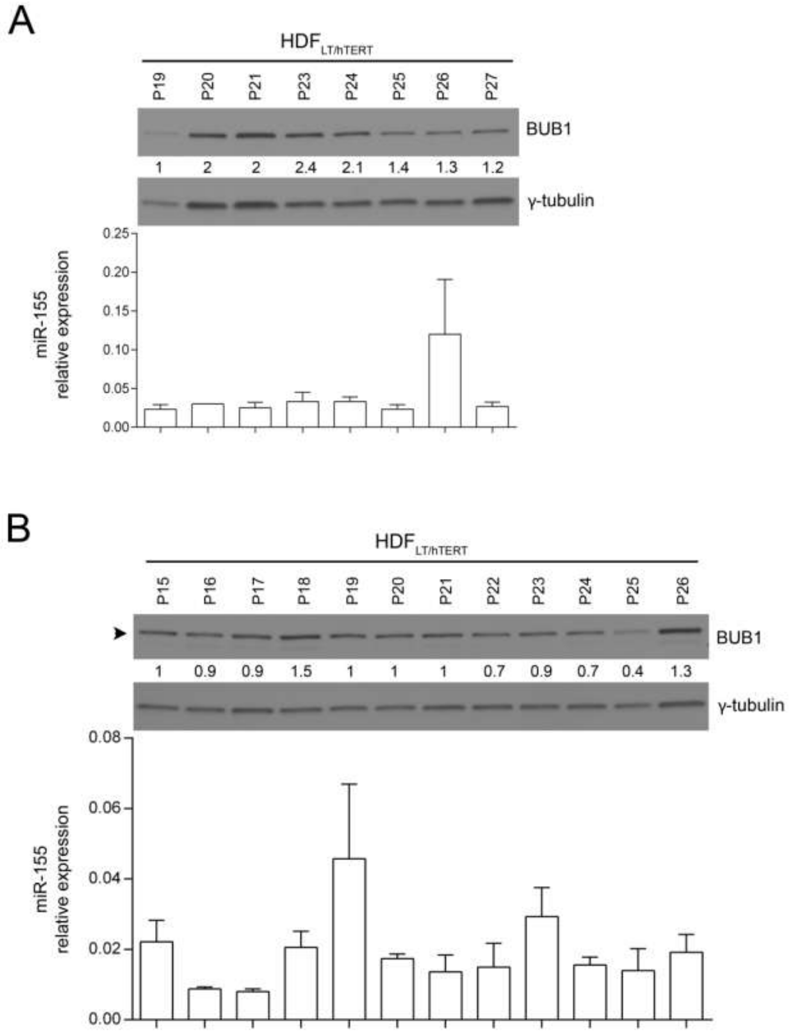

Figure S1. (**A**) and (**B**) western blot analysis of BUB1 protein expression during the immortalization passages of $HDF_{LT/hTERT}$ cells in two independent experiments. *MiR-155* relative expressions are shown. Data are means ± SD of three technical replicates.

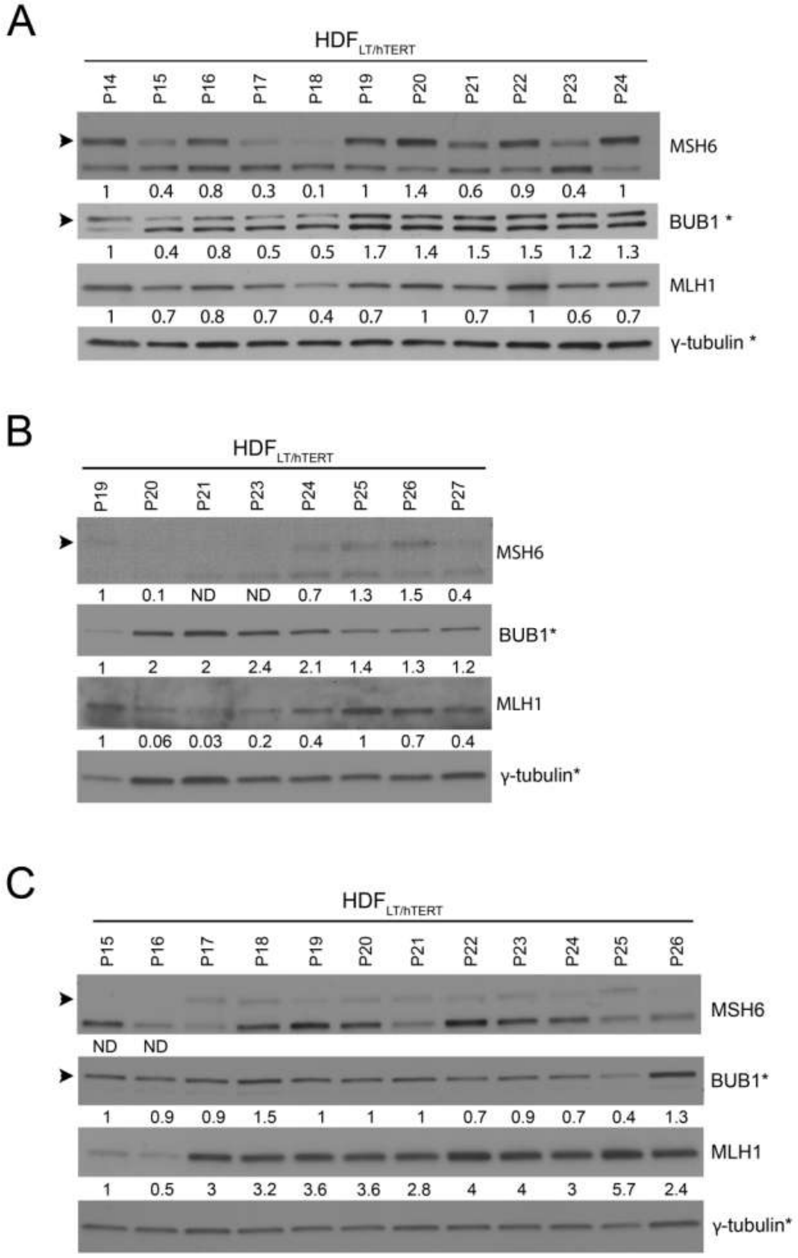

Figure S2. (**A**,**B**) and (**C**) Western blotting analysis of MSH6 and MLH1 proteins during the immortalization passages of $HDF_{LT/hTERT}$ cells in the three independent experiments showed in figure 1D and supplementary figure S1. (*) western blot pictures and analysis (BUB1 and γ-tubulin) already presented in Figure 1D and in supplementary figure S1 of the manuscript.

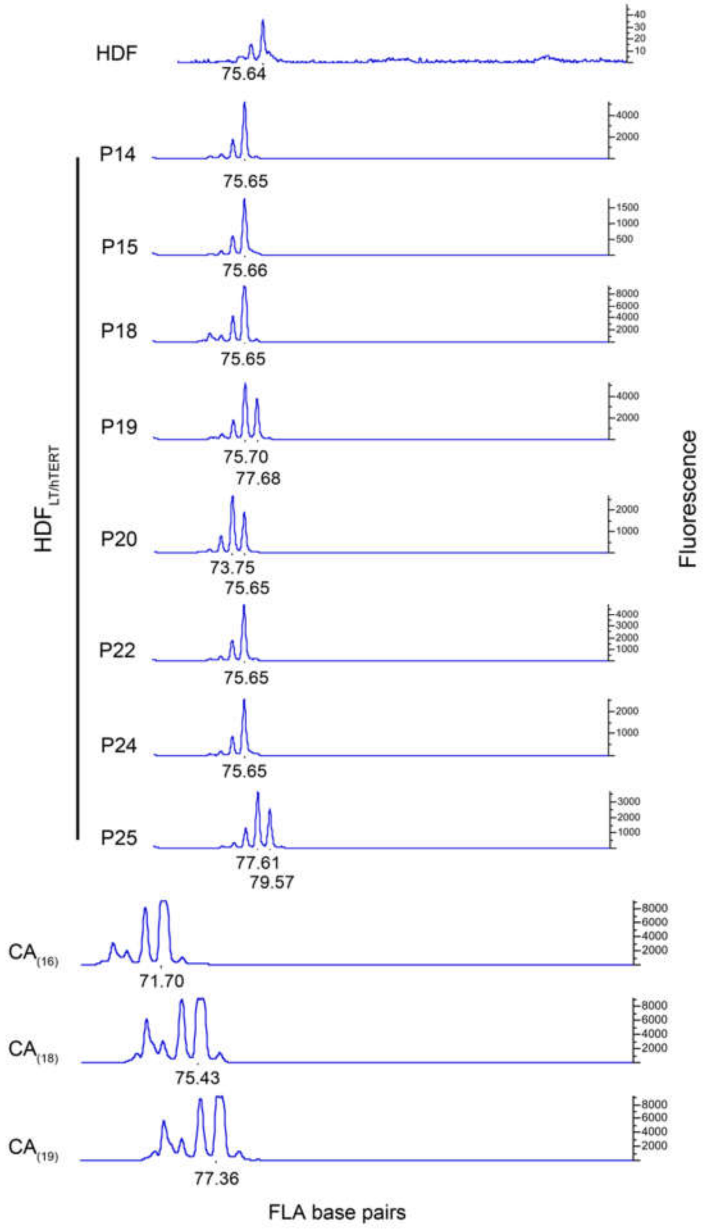

Figure S3. Fragment length analysis (FLA) of the D2S18888 polymorphic marker in immortalized HDF cells.

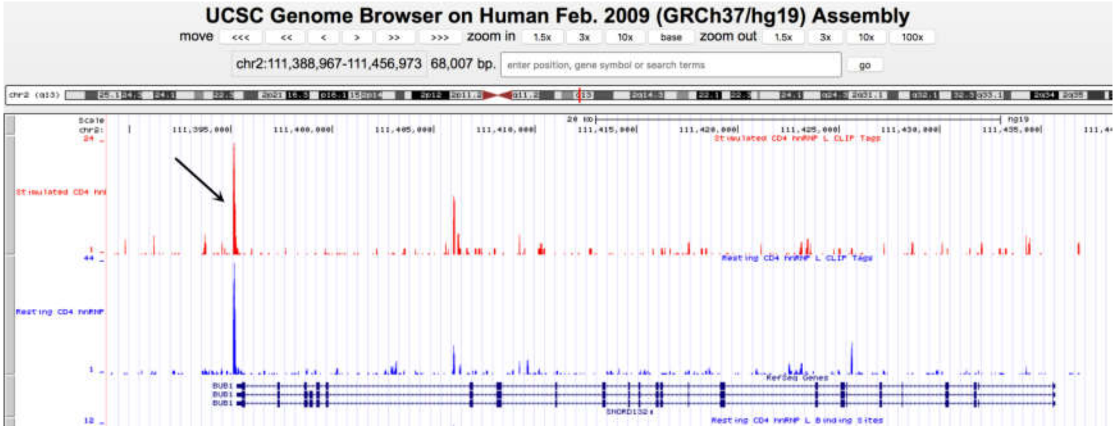

Figure S4. Screen shot of the UCSC Genome Browser showing the HNRNPL binding sites on the *BUB1* gene (BUB1 exons and UTRs are represented by blu boxes) in CD4 positive cells. Black arrow indicates the HNRNPL binding on the CA repeats of the *BUB1* 3′UTR, based on data provided by Shankarling et al. [1].

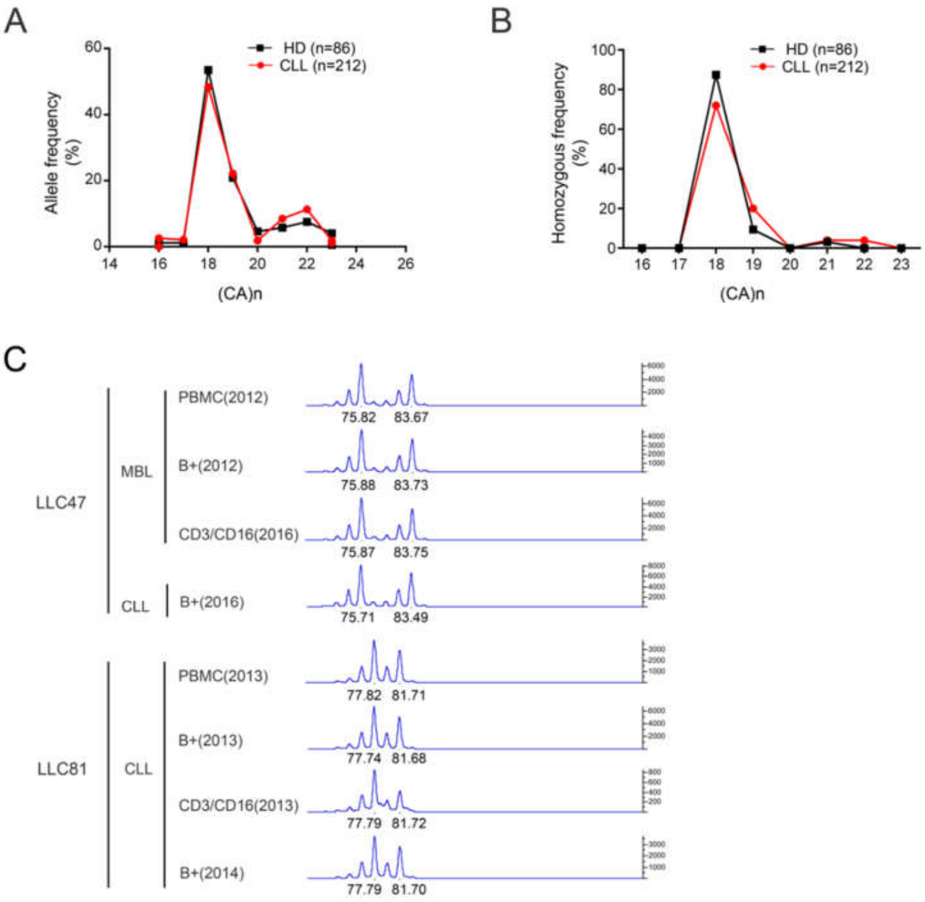

Figure S5. (**A**) total allele frequency and (**B**) homozygous frequency of the D2S18888 polymorphic marker length in DNA samples from healthy donors (HD) and CLL patients, analyzed by fragment length analysis. (**C**) Electropherograms of the D2S1888 amplicon in DNA from total (PBMC), normal (CD3/CD16 positive cells) and leukemic (B+) cell fractions from two patients (LLC47 and LLC81) at different stages of disease. MBL, monoclonal B-cell lymphocytosis.

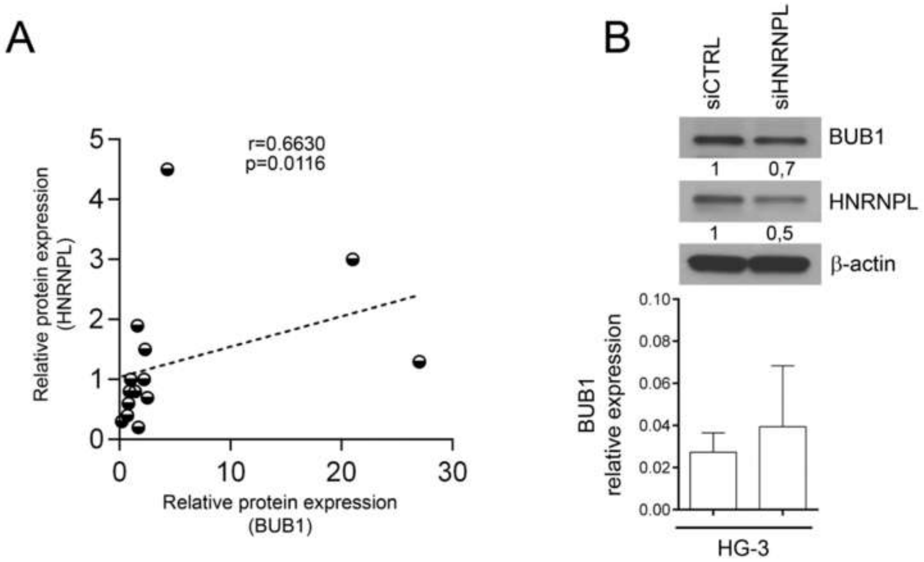

Figure S6. (**A**) Graphical representation of the correlation analysis between BUB1 and HNRNPL protein expression in the CLL patients listed in figure 3. For each patient, the relative protein expression is related to the first time point value (first time point = 1). Analysis of correlation was performed by non-parametric Spearman's correlation, two tailed. (**B**) Western blots of BUB1 and HNRNPL proteins (120 kDa) and BUB1 mRNA relative expression in HG-3 cells transfected with either siRNA control or siRNA of HNRNPL for 72 h. Densitometric values normalized to β-actin protein expression are reported. Data of BUB1 mRNA are means ± SD of three technical replicates.

Reference

1 Shankarling, G.; Cole, B.S.; Mallory, M.J.; Lynch, K.W. Transcriptome-wide RNA interaction profiling reveals physical and functional targets of hnRNP L in human T cells. *Mol. Cell. Biol.* 2014, 34 71–83.

© 2019 by the authors. Licensee MDPI, Basel, Switzerland. This article is an open access article distributed under the terms and conditions of the Creative Commons Attribution (CC BY) license (http://creativecommons.org/licenses/by/4.0/).
